# Bio-active peptides (Biogro™) supplementation improves work capacity during short-term resistance exercise in men

**DOI:** 10.1186/1550-2783-11-S1-P26

**Published:** 2014-12-01

**Authors:** Carleigh H Boone, Kyle S Beyer, Jeffrey R Stout, Jay R Hoffman, Maren S Fragala, David H Fukuda

**Affiliations:** 1 University of Central Florida, Orlando, Florida, USA

## Background

Inadequate recovery between training sessions leads to fatigue and imposes a hindrance on exercise performance. Bio-Active Peptides (BAPs) supplementation has been shown to reduce recovery time between strenuous exercise bouts and improve work capacity during subsequent bouts in athletes. However, few investigations have explored the efficacy of BAP supplementation on early performance adaptations in previously untrained men.

## Methods

Maximal dynamic strength (kg) of leg press (LP), leg extension (LE), chest press (CP), and low row (LR) exercises was assessed of eighteen untrained men (22.3 ± 2.8y; 25.3 ± 5.5kg•m-2) who volunteered to participate in this study. Lower body strength was assessed via one-repetition maximum (1RM) testing; upper body strength was estimated as [repetition weight/(1.0278-0.0278)(reps)]. Participants were randomized into either a training only group (RT) or training + BAP supplementation (COL) (3g/day for 28 days). All participants completed four weeks of training (3 days/week; 12 total sessions) wherein each exercise was performed at 80% 1RM for 3 × 8-10 reps. Daily training volume was calculated as weight × reps. Changes in daily training volume were analyzed using magnitude-based inferences, calculated from 90% confidence intervals. Consent to publish the results was obtained from all participants.

## Results

Analyses revealed that BAP supplementation provided an 80.8% chance of increasing total training volume from Week 1 to Week 2 compared to the RT group. From Week 1 to Week 3, BAP supplementation had a 50.9% chance of increasing total training volume compared to the RT group. BAP supplementation provided a 60.7% and 54.0% chance of increasing total training volume from Week 1 to Week 4 and Week 2 to Week 3, respectively. From the first to last training sessions, BAP supplementation displayed a 90.9% chance of increasing total training volume compared to the RT group.

## Conclusions

Untrained individuals are especially susceptible to fatigue and soreness following exercise and often warrant extended recovery time at the onset of a training program. The current results suggest that supplementing with BAPs during the early phase of a training program may aid in reducing the recovery time between bouts of strenuous exercise. By encouraging faster recovery, BAP supplementation may promote earlier adaptations to training (i.e., increased strength and training volume) as compared to training without supplementation.

